# A design and optimization of a high throughput valve based microfluidic device for single cell compartmentalization and analysis

**DOI:** 10.1038/s41598-021-92472-w

**Published:** 2021-06-21

**Authors:** Jonathan Briones, Wilfred Espulgar, Shohei Koyama, Hyota Takamatsu, Eiichi Tamiya, Masato Saito

**Affiliations:** 1 Graduate School of Engineering, Osaka University, Suita, Osaka 565-0871 Japan; 2 Department of Respiratory Medicine and Clinical Immunology, Graduate School of Medicine, Osaka University, Suita, Osaka 565-0871 Japan; 3 AIST PhotoBIO-OIL, Osaka University, Suita, Osaka 565-0871 Japan; 4 The Institute of Scientific and Industrial Research, Osaka University, Ibaraki, Osaka 567-0047 Japan

**Keywords:** Biomedical engineering, Chemical engineering, Cancer, Lung cancer, Bioanalytical chemistry, Lab-on-a-chip

## Abstract

The need for high throughput single cell screening platforms has been increasing with advancements in genomics and proteomics to identify heterogeneity, unique cell subsets or super mutants from thousands of cells within a population. For real-time monitoring of enzyme kinetics and protein expression profiling, valve-based microfluidics or pneumatic valving that can compartmentalize single cells is advantageous by providing on-demand fluid exchange capability for several steps in assay protocol and on-chip culturing. However, this technique is throughput limited by the number of compartments in the array. Thus, one big challenge lies in increasing the number of microvalves to several thousand that can be actuated in the microfluidic device to confine enzymes and substrates in picoliter volumes. This work explores the design and optimizations done on a microfluidic platform to achieve high-throughput single cell compartmentalization as applied to single-cell enzymatic assay for protein expression quantification. Design modeling through COMSOL Multiphysics was utilized to determine the circular microvalve’s optimized parameters, which can close thousands of microchambers in an array at lower sealing pressure. Multiphysical modeling results demonstrated the relationships of geometry, valve dimensions, and sealing pressure, which were applied in the fabrication of a microfluidic device comprising of up to 5000 hydrodynamic traps and corresponding microvalves. Comparing the effects of geometry, actuation media and fabrication technique, a sealing pressure as low as 0.04 MPa was achieved. Applying to single cell enzymatic assay, variations in granzyme B activity in Jurkat and human PBMC cells were observed. Improvement in the microfluidic chip’s throughput is significant in single cell analysis applications, especially in drug discovery and treatment personalization.

## Introduction

Microfluidic technology has become a popular choice for micro-total analysis systems by providing high precision of liquid manipulation that conventional bench-top approaches are unable to do^1,2^. Many research works have demonstrated its advantages in having fast isolation speed and high efficiency of data acquisition. These platforms are capable of spatial control of liquid composition, cell and cell environment manipulation, and single cell handling and quantification of secreted molecules^3–5^. Thus, microfluidic platforms are an important tool for single cell studies as it allows the identification of rare subsets and resolution between cells with same expression patterns. This has become an important tool for single cell biological research taking advantage of massive parallel processing, streamlining of complex protocols, and enabling high-throughput biological experiments^3,5^.

For experiments that manipulate large number of cells simultaneously and independently, a high-throughput platform that is capable of large-scale integration and integration of numerous analytical standard operations is needed. There are several microfluidics techniques that can be utilized for single cell research like droplet microfluidics, microwells and hydrodynamic trapping, etc. However, in enzymatic activity assay that aims to quantify extracellular protein secretions (i.e. granzyme B protease) from each cell, a platform that is capable of compartmentalization, on-demand media exchanges for washing and reagent introduction, and real-time monitoring is needed. Here, a microvalve-based device is a better choice as compared to other techniques^6,7^. There are several types of actuation mechanisms for microvalves, however this work focused on normally open pneumatic valves (i.e. Quake valves). A valve is made of a control channel that overlaps a flow channel and is separated by a thin membrane. Generally made of polydimethylsiloxane (PDMS), an external gas or liquid source that is connected to the control line provides pressure that actuates the valves, which cause the thin membrane to deflect and block or close the flow channel^7,8^. Nonetheless, the throughput of this technique is limited by the number of available compartments that can be sealed by actuated valves below critical pressure.

Meier et al. made use of 540 microfluidic PDMS membrane valves and Armbretch et al. used 1026 circular membrane valves and corresponding microchambers in their respective research works^9,10^. Both works applied about 0.20 MPa as actuation pressure to seal the microchambers and isolate the samples. The group of Kellogg et al.^11^ applied ≈ 0.17 MPa (25 psi) in their control line to isolate single cells in 96 chambers. Tong et al. needed ≈ 0.14 MPa (20 psi) to switch the valves (4 × 4 addressable ports) to a closed state^12^.

With the advancements in the fabrication techniques of microchip-based screening devices, which are capable of nanoliter-scale liquid-handling, the throughput of single cell experiments also increases. High throughput screening has been defined as the analysis of more than 10^3^ data points tested in parallel or very rapidly in succession^13,14^. For a valve-based microfluidic device, increasing the number of chambers that can compartmentalize each cell to more than 10^3^ is advantageous as it would allow greater representation of the sample population under investigation, which can further probe the significance of heterogeneities. Here, the amount of external pressure, delivered to the PDMS membrane during hydraulic actuation, is critical as higher values could put the thin membrane at risk of rupturing, breaking of the bonds between the polymer layers, or the occurrence of leaks and air permeation to the flow channel. In a high-dense network with multiple valves linked in series, an increase in the actuation pressure have been reported across a network of connected valves^15,16^. Increasing the number of linked valves, i.e. 10^3^, and channel length have been reported to result to higher gas release, due to gas absorption and PDMS permeability, because of increased PDMS wall surface and gas leakage at tubing connections^16^. One solution to compensate the increase in actuation pressure is to increase the contact area of the valve layer and the flow or bottom layer^15,16^. However, considering the need for high throughput, particularly the number of microchambers viewable in a single frame, the target cells should be positioned as close as possible. Thus, other parameters such membrane deformability, valve geometry, actuation media, and the like should be altered and optimized to decrease the actuation pressure.

In this work, we sought to increase the number of microchambers in an array of a microfluidic chip that can be opened or closed with circular microvalves. Using COMSOL Multiphysics, we investigated the effect of the geometry and dimensions of the valve design to the sealing pressure. The analysis of the multiphysics system model aims to reduce the value of the sealing pressure in real applications. These results were used to fabricate a microfluidic device with up to 5000 hydrodynamic traps and control microvalves via photolithography and soft lithography techniques. The effects of different actuation media, fabrication technique, and PDMS ratio were also investigated to determine the lowest achievable sealing pressure within the constraints of the chip. The said device was later applied to perform a high-throughput single cell enzyme activity assay to profile the granzyme B (GrB) expression of each immune cell.

## Results and discussion

### Model implementation and valve design optimization

**Figure 1:**
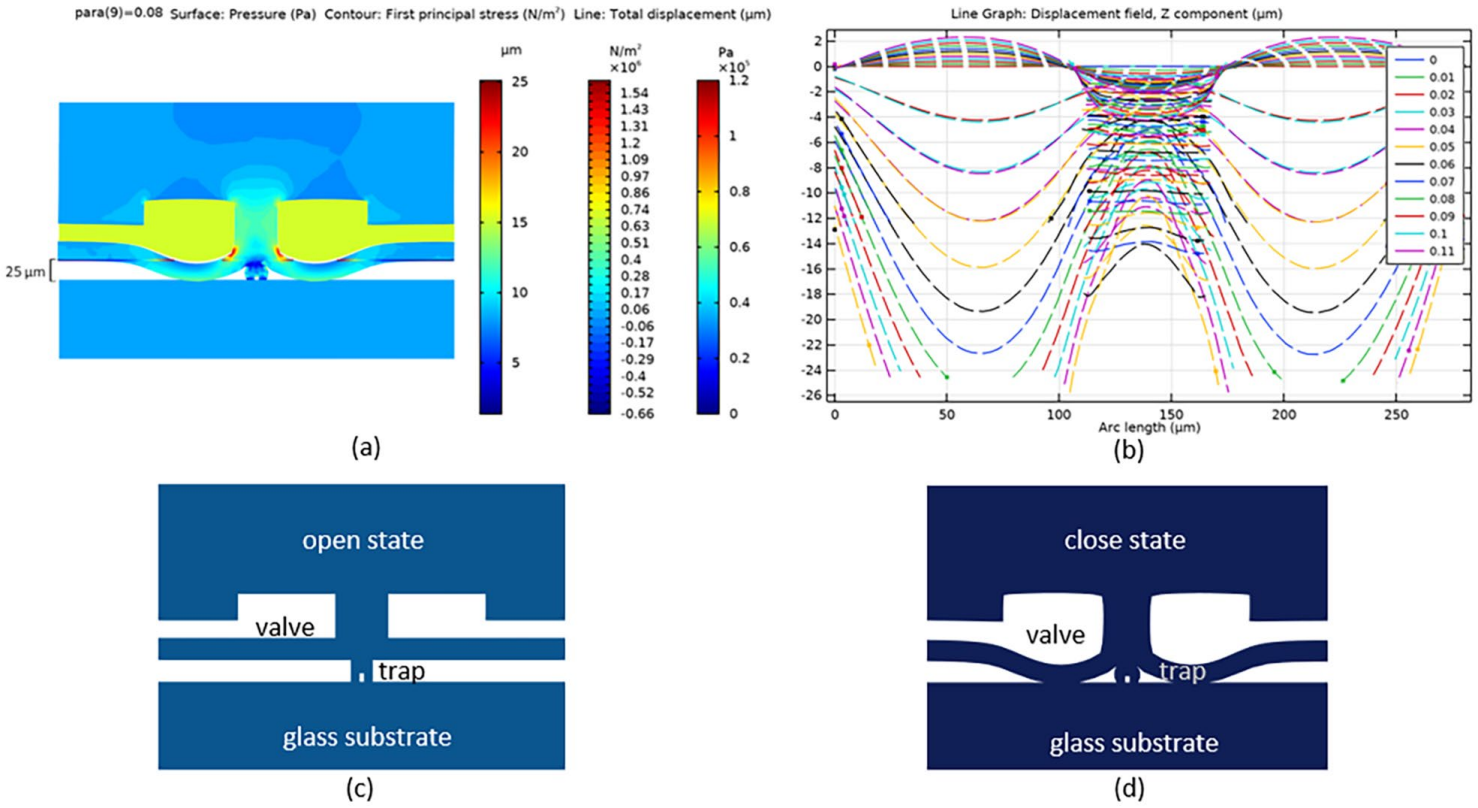
Multiphysics simulation of the unit valve’s (R = 140 µ, h = 50) actuation. (**a**) 2D plot of the middle cut plane indicating membrane deflection, resulting surface pressure and first principal stress, (**b**) line graph of the displacement field at various applied pressure (MPa) across the arc length, (**c**) illustration depicting an open state and (**d**) close state. Figures were generated using COMSOL Multiphysics 5.5.

Figure 1 shows one of the simulations done on the microvalve unit. This 2D plot result of the middle cut plane models the valve actuation and the field displacement (z component) when a pressure of 0.08 MPa was applied to deflect the elastomer membrane across the flow channel with a height of 25 µm. This example was chosen to show how the sealing pressure was determined during actuation to cause the membrane to be displaced to more than the flow channel’s height.

The effect of the outer radius (R) of the circular valve model and the applied pressure, to the membrane displacement was compared and is summarized in Fig. 1b. The modeling showed that with a valve height is 25 µm, a valve radius of 80 µm requires a pressure of 0.33 MPa to deflect the membrane and create a sealed chamber. Increasing R to 100  µm decreases the sealing pressure to 0.21 MPa. Meanwhile an R of 120 µm needs to reach 0.13 MPa. Further increasing R to 140 µm lowers the sealing pressure to 0.08 MPa. When the valve height was increased to 50 µm, a decrease in sealing pressure was computed. A sealing pressure of 0.29 MPa, 0.19 MPa, 0.12 MPa, and 0.075 MPa resulted from an R of 80 µm, 100 µm, 120 µm, and 140 µm, respectively.

**Figure 2:**
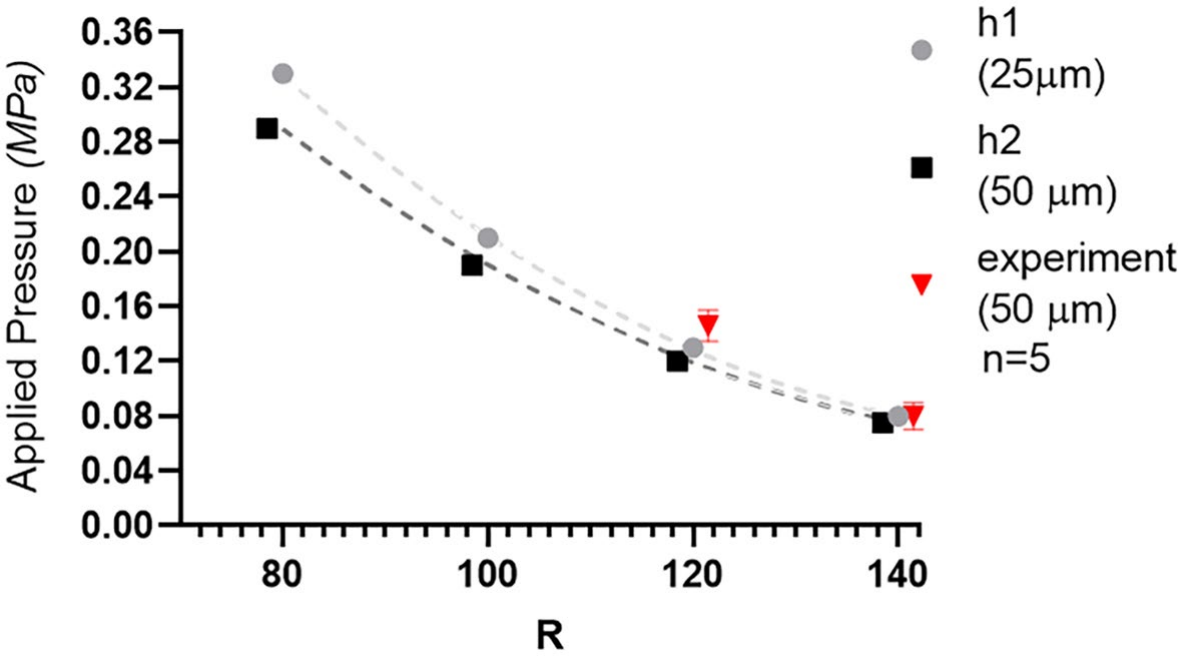
Relationship of the valve radius R and height h to the sealing pressure. Graph created using GraphPad Prism 8.4.3.

It can be seen in Figs. 1 and 2 that, the displacement, for different loads, and the corresponding sealing pressure varies with R. Curve fitting by nonlinear regression (second order polynomial) shows a good fit with an R-square of 0.9999 (99.99%) for h1 and 1 (100%) for h2. It can also be seen that a higher h (h1 vs h2) resulted to a lower sealing pressure, although this difference decreases as the R increases.

From these results, we fabricated a microfluidic chip with arrays of microvalves with height of h2 (50 µm), and R of 120 µm and 140 µm. One control line inlet is connected to about 750 circular valves. There are 3 interconnected inlets on each side of the device. We tested the actuation of these valves by applying air pressure at the control inlet. The flow layer was filled with a FITC dextran fluorescent solution and the fluorescence intensities were measured with and without actuation. A sealed chamber is determined when the intensity in the valve area is the same as the background or no fluorescent solution.

As shown in Fig. 2, the microfluidic valves with R of 120 µm can be sealed with the lowest pressure of 0.13 MPa (mean, $\stackrel{-}{x}$ = 0.14 MPa, std. dev. = 0.011) as compared to the computational value of 0.12 MPa. On the other hand, experimental results for the valves with R = 140 µm showed a sealing pressure of 0.07 MPa ($\stackrel{-}{x}$ = 0.08 MPa, std. dev. = 0.007) as compared to the simulation value of 0.075 MPa. The R was limited to a maximum value of 140 µm as further increasing it would result to a greater separation distance between hydrodynamic traps, which lowers the trap occupancy rate, and increases overall microfluidic chip size.

**Figure 3:**
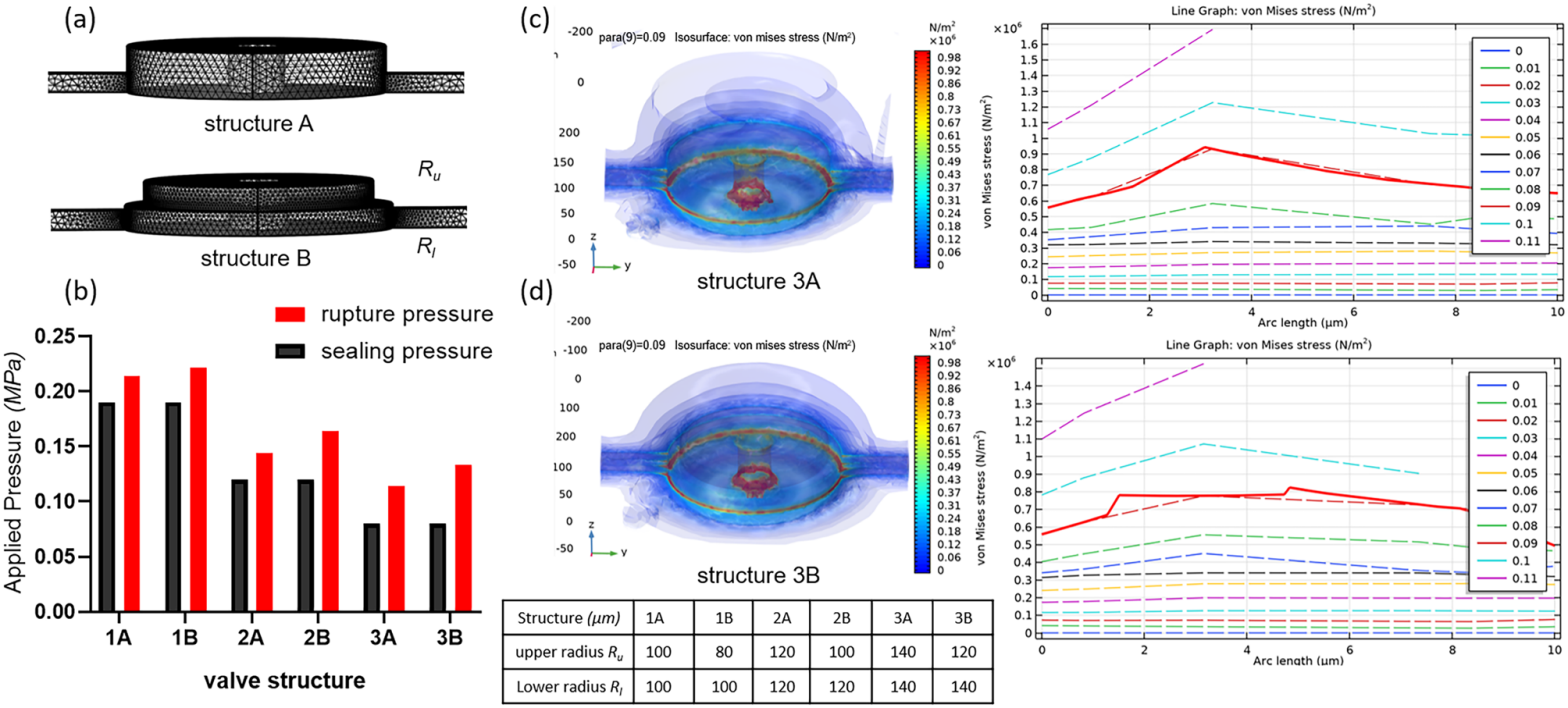
Effect of the valve structure on sealing pressure and rupture pressure/computation failure (**a**) mesh geometries of valve structures (**b**) comparison of the sealing and rupture pressures by valve structures (**c**) 3D modeling and line graph of the von Mises stress in structure 3A and (**d**) structure 3B. Figures were generated using COMSOL Multiphysics 5.5 software.

Using the COMSOL Multiphysics simulation, we compared the effect of different valve structures to the sealing pressure and the point of computation failure (rupture pressure), shown in Fig. 3. The failure mode termed here as rupture pressure is defined as the highest amount of applied pressure the microchamber unit can hold before compartmentalization fails, which can be due to membrane rupture, layer-layer bonding failure, or both. Valve structures A and B (Fig. 3a) both have a height of 50 µm and lower radius *R*_*l*_. However, structure B’s upper radius *R*_*u*_ is smaller by 20 µm, creating a step or bell like figure. The smaller top structure was chosen with the aim of reducing the material strain in the membrane as well as lower the total volume of the valve. This aspect ratio of smaller top and bigger bottom is also assumed to avoid membrane collapse.

Simulation results showed that both valve structures A and B achieved the same sealing pressure at different R (Fig. 3b). However, when it comes to the maximum pressure that the unit can hold before failure, structure B reaches a higher value. This shows that structure A reaches a mode of failure first as the load increases. One difference among the two designs is the total volume of the valve unit, smaller for B. Figures 3c and 6d shows the von Mises stress of the solid at a cut line defined at the circular valve geometry of structures 3A and 3B, respectively. Structure 3B (fails at 0.133 MPa) was computed to have relatively lower von Mises stress value, at sealing pressure loads of 0.08 to 0.11 MPa, than 3A (fails at 0.114 MPa).

The von Mises stress is a failure criterion used to predict the yielding of the isotropic material under the pressure load, in which said yielding starts when the critical value for the elastic energy distortion is reached^17^. A higher von Mises value would mean that the material is near the yield point. In the case of the valve unit, structure A values were seen to be bigger than structure B. A smaller von Mises stress is better for a more durable device, which led us to choose structure 3B in the fabrication of the high-throughput microfluidic chip.

The von Mises stress failure criterion is related to strain energy density which tells the failure of the material once the distortional strain energy exceeds a critical value, which in turn depends on the design parameters^18^. The strain energy density function (*W*) or the stored energy density refers to the energy stored in the material per unit volume of the original geometry as a function of strain at that material point. This is defined by the following equation^19^:

1 $$\begin{aligned} W & = f\left( {I_{1} ,~I_{2} ,~I_{3} } \right) \\ & = f\left( {\left( {\lambda _{1}^{2} + \lambda _{2}^{2} + \lambda _{3}^{2} } \right),~\left( {\lambda _{1}^{2} \lambda _{2}^{2} + \lambda _{2}^{2} \lambda _{3}^{2} + \lambda _{3}^{2} \lambda _{1}^{2} } \right),\left( {\lambda _{1}^{2} \lambda _{2}^{2} \lambda _{3}^{2} } \right)} \right) \\ \end{aligned}$$

where *I*_1_,* I*_2_, *I*_3_ are strain invariants of Green deformation tensor. Individual strain invariants are a function of the principal stretch ratios (*λ*_*i*_,* i* = 1, 2, 3).

Lower values of strain energy density in structure 3B is possibly due to its smaller valve cavity volume, as reflected by its smaller *R*_*u*_, and the volume of pressurized gas that it holds. The low values of strain energy density would mean low risk of material failure. The shear stress and stored energy density for different loads between structures A and B are shown in Fig. S8 of the supplementary material.

### Hydrodynamic trapping

As previously mentioned, the maximum R of the valve that was included in the modelling was kept at 140 µm so as to keep the distance between adjacent mechanical traps around 300 µm. This limitation was imposed to avoid further decrease in the single cell trap occupancy rate.

The previous version of the fabricated device, reported elsewhere^20^, had 1080 traps and corresponding chambers. In order to have a higher throughput and better representation of the sample population, the microfluidic chip was expanded up to 5000 traps/chambers. To determine how many single cells can be mechanically trapped, we allowed 100 µL of Jurkat cells at a concentration of 10^6^ ml^−1^ to flow in the flow channel at different flow rates using a syringe pump.

**Figure 4:**
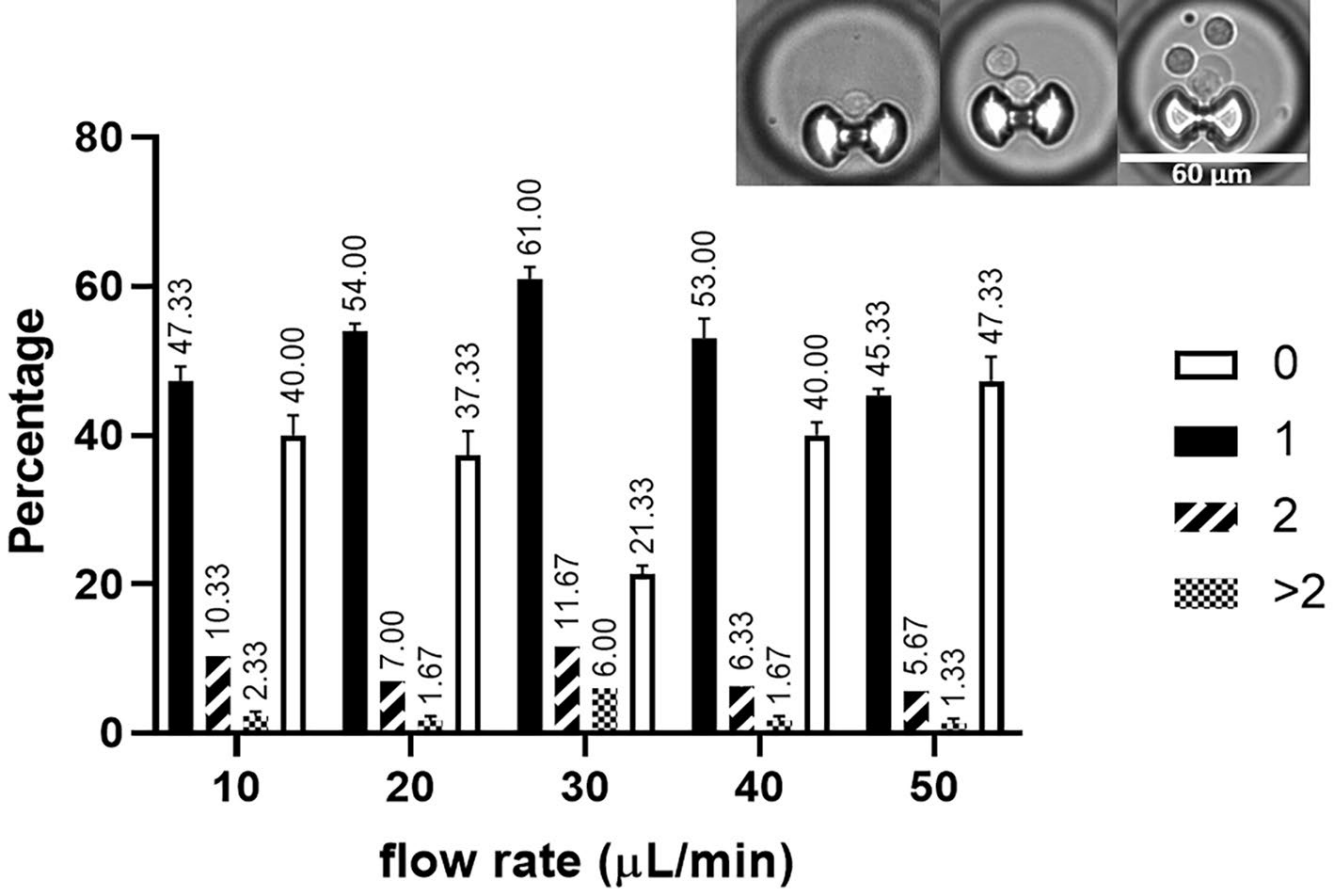
Percentage of trap occupancy at different flow rates. The percentage of traps with single capture is shown as a black solid bar, double capture as diagonal-patterned bar, and traps with more than 2 cells is shown as chequered bar. Only traps with single cell were analyzed. Inset, representative images of the captured cells. Graph created using GraphPad Prism 8.4.3.

Shown in Fig. 4 is the result of the cell trapping test that was conducted. At 30 µL min^−1^ flow rate, the chip attained 61% single cell trap occupancy, about 12% had double-cell occupancy and 6% with more than 2 cells. At 20 and 40 µL min^−1^ flow rates, about 54% and 53% of the traps were occupied by one cell. The double cell occupancy is at 7% and 6%, respectively, while ≈ 2% have more than 2 cells. The single cell trap occupancy further decreased to about 47% and 45% at flow rates of 10 and 50 µL min^−1^, respectively. 10% and 6% contain two cells while 2% and 1% contain more than two cells for respective flow rates of 10 and 50 µL min^−1^. On the other hand, increasing the concentration and volume of the cell suspension introduced into the chip can increase the trap rate and reach up to 80% trap occupancy. The flow rate of 30 µL min^−1^ was later on used in the assay experiment and traps with more than 1 cell were excluded from the data evaluation process.

### Structure reinforcement

Because of its viscoelasticity, thermosetting and inert properties, PDMS is a popular choice in the fabrication of microfluidic devices. This flexible silicone-based organic material is a viable hyperelastic material for COMSOL Multiphysics simulation, which helps explain the effects of the design parameters in valve actuation and compartmentalization^19^. Similarly, the fabricated microfluidic device is made up mostly of PDMS with a density of 0.95^29^ ρ/g cm^−3^ and a Young’s modulus, E, that is thickness dependent because of the reordering of the polymer chains to form cross-linked networks^21^. The degree of cross-linking is related to the elastomer base-curing agent ratio. In this work, the flow and control layers are composed of a 10:1 and 5:1 ratio, respectively.

The elastic modulus, E, in MPa can be expressed as a function of the PDMS base/curing agent weight ratio, N, as^22^:

2 $$E = 20\,{\text{MPa}}/{\text{N}},$$

Wang et al. measured E for a 5:1 ratio to be about 3.58 MPa while the 10:1 ratio is about 2.63 MPa^14,22^. Increasing the amount of the curing agent, such as the case of 5:1 ratio, results to a stiffer PDMS. Desai et al. modeled membrane stiffness, which can be applied to rectangular or circular membrane valves, and is given by^21,23^:

3 $$k = K_{{bilayer}} K_{{post}} \frac{{K_{{MS}} }}{{K_{{AR}} }}~\left( {K_{{shape}}^{1} \frac{{E_{{bm}} t_{m}^{3} }}{{D^{4} }} + K_{{shape}}^{2} \frac{{st_{m} }}{{D^{2} }}} \right),$$

where *k* is static membrane stiffness per membrane unit area, $E_{{bm}}$ is biaxial stiffness, *t*_*m*_ the total thickness of the membrane, *D* the membrane diameter or length, *K*_*bilayer*_ is bilayer setup factor, *K*_*post*_ cylindrical support post factor, *K*_*MS*_ membrane stress effect (when membrane deflection exceeds half its thickness), *K*_*AR*_ aspect ratio factor, *K*_*shape*_ shape of channels factor (rectangular or circular), and *s* is residual stresses^21^.

Generally, PDMS is a soft elastomer material that is susceptible to buckling under pneumatic or hydrostatic loading. At high pressure inputs, the valve chamber walls undergo large deformations, especially on the thin membrane walls (i.e. bottom of the valve cavity) as shown in Fig. 1b. Although above the valve channel is a thicker and stiffer control layer, change in geometry (displacement field) is observable as a result of the valve actuation.

**Figure 5:**
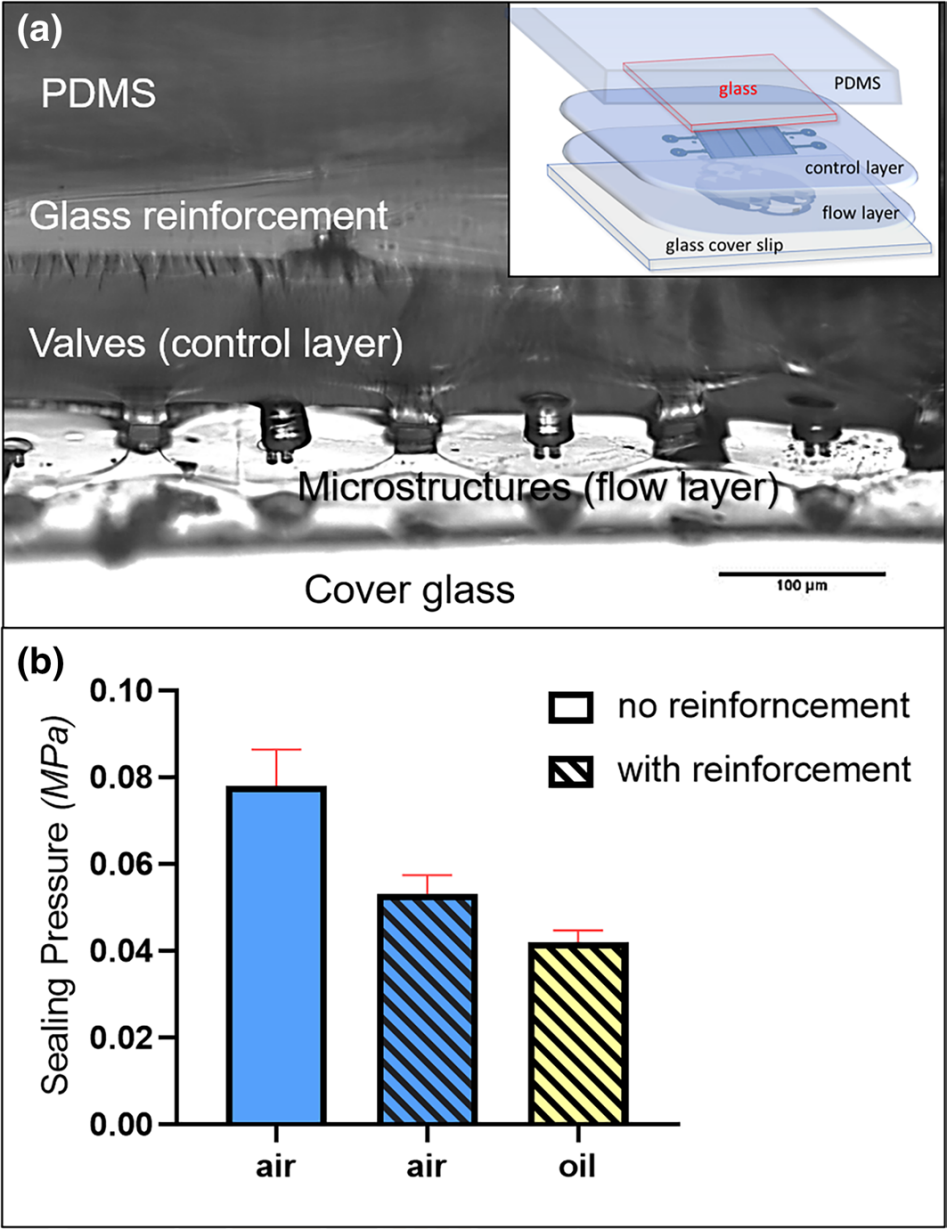
Reinforcement of the control layer via addition of thin glass ≈ 100 µm above the PDMS microvalves (**a**) cross-section image of the microfluidic device, inset: by-layer composition illustration (**b**) comparison of the sealing pressures at varying conditions (n = 5).

In order to reduce the volume expansion on top of the valve cavity, consequentially reduce deformation and strain on the portions of the structure that is not critical for bending, a custom fabrication of adding a thin glass sheet as reinforcement (Matsunami coverglass; 0.13–0.17 mm thickness) about 100 µm on top of the control layer valves was done and is shown in Fig. 5a. This combination of materials results to an even stiffer top component. This can result to a smaller deformation or volume change.

The reduction of the expansion volume of non-critical actuator sections can increase the speed at which the softer membrane at the bottom bends during actuation. This in turn can reduce the sealing pressure needed to close thousands of chambers in array^24^. At the same time, the reduced deformation and sealing pressure can result to lower material strain and improved durability.

With the optimized design structure and fabrication reinforcement, an actuation test was done to the array of microvalves to determine the working pressure needed to seal all the microchambers. Without the glass reinforcement, the lowest pneumatic sealing pressure achieved was 0.07 MPa ($\stackrel{-}{x}$ = 0.08, std. dev. = 0.008) as shown in Fig. 5b. With the addition of the glass top, the sealing pressure was reduced to 0.05 MPa ($\stackrel{-}{x}$ = 0.053, std. dev. = 0.004). We also explored the effect of the actuating media by substituting air with mineral oil (Sigma Aldrich M5904) and found that the sealing pressure was further reduced to 0.04 MPa ($\stackrel{-}{x}$ = 0.042, std. dev. = 0.003).

This reduction in the working pressure when oil was used as actuation medium can be due to the suppression of permeability of the thin PDMS membrane in the microchambers. PDMS is permeable to gases and this can cause a problem to microfluidic devices with valve actuation operated at high operating pressures and longer periods of time^25^. A potential test failure results from the bubbles arising inside the microfluidic channels, especially for long experiments and cell analysis (hours to days)^26^. Free volumes allowing gas diffusion inside are due to the high flexibility of the Si-O chains in silicones. Here, the diffusion happens with the migrations of gas molecules from one hole to another and eventually evaporating out of the thin PDMS membrane^26^. In our experience, pneumatic valve actuations during assay experiments for many hours can result to gas bubble formation inside the fluidic channels, which ruins the assay protocol (i.e. media exchange) or the entire experiment itself. During this period of continuous actuation for more than 3 h at applied pressures above 0.12 MPa, where there is a constant flow of washing buffer from the inlet, through the main flow channel, and out to the outlet connected to a syringe pump, bubble formation is observed emanating either from the area near the edge of the inflated valve or from within the chamber (similar to supplementary video S10). The actuation of the membrane valves can result to displacement of surrounding liquid, uniformly distributed in all directions but this effect is offset by the flux of reagent flowing in the channel. Thus, for long actuation time, gas is not the best choice as actuation medium. Other media such as water (i.e. deionized water) and mineral oil maybe a better choice.

Using microfluidic device with water filled control channels or microvalves, effects of evaporation can be observed if used for long incubation times. This decrease in the actuating liquid consequently sometimes results to some water-air interface or bubbles in the valves which are seen during image acquisition. Although this can be resolved with the use of tubing connected to water reservoir, it creates clutter and consumes time to set-up and disconnect when the microfluidic devices are moved from the microscope to the incubator for several times at varying incubation times, before, during, and after assay experiments. For this reason, we preferred the use of mineral oil as the alternative actuation medium for a ready to use chip in cell experiments.

For this material, the relationship between the volumetric flow rate *q*, permeability coefficient *P*, membrane thickness *t*, valve radius *R*, and pressure difference Δp is given by^27^:

4 $$P = \frac{{qt}}{{\pi R^{2} \Delta p}},$$

The equation above (Eq. 4) tells that the volumetric flow *q* through the PDMS membrane is inversely proportional to the thickness, which is significant for thin layers. The thickness of the membrane, which dictates its flexibility and degree of deflection, is also crucial for the valve functionality. With oil filling the valve cavities, a barrier between the pressurized gas and permeable membrane is created. In effect, this suppresses the gas permeability and reduce the sealing pressure.

The use of oil in valve actuation is also seen advantageous for devices with high density of network channels and high valve population since pressure losses from pneumatic actuation, due to gas release from gas absorption and PDMS permeability (as the PDMS wall surface is increased), are avoided or reduced^16^.

In fluid mechanics, this can be represented by the following^16^:

5 $$P_{{act}} = ~P_{0} + \left( {q_{l} + q_{p} } \right)/S$$

where *P*_*act*_ or actuation pressure is the total pressure in the control channels, *P*_*0*_ is the critical pressure of the pump, *q*_*l*_ is gas leakage flow rate at tubing connections, *q*_*p*_ is rate of gas release from gas absorption and permeability in PDMS, and *S* is pumps’ pumping rate. Equation 5 tells that increasing the valve population, the channel length, and consequently the PDMS wall surface leads to increase in gas leakage and gas release. In order to have a more reliable operation, the gas leakage and release need to be reduced. The reduction of the actuation pressure to 0.04 MPa for a 5000-chamber array is an important initial step as it gives more room for any increase in pressure when the throughput is further increased (i.e., 10,000, 20,000, etc.).

### PDMS off-ratio and membrane thickness

Initially, the fabricated microfluidic chip following the simulation results made use of a 10:1 PDMS ratio for the flow layer and 5:1 ratio for the control layer. The said ratios were used in congruence with the device used for single cell granzyme B profiling of model cells and PBMC samples reported elsewhere^20^. However, the said ratio for PDMS-PDMS bonding is not the optimized ratio to yield a stronger bonding for multi-layer softlithography. Using the optimal off-ratio bonding, it has been reported to hold up to 0.496 MPa (72 PSI), which is above the normal microfluidic operating range 0.138–0.310 MPa (20–45 PSI)^28^. A more optimal ratio is 20:1 for flow layer and 10:1 or 5:1 for control layer. In our actuation test using fabricated microfluidic device with the said ratios, both devices with 20:1 flow layer and 10:1 control layer, and 20:1 flow layer and 5:1 control layer were able to hold > 0.24 MPa (limit of pump) of applied pressure as compared to the previous device which reached up to 0.18 MPa before failure as shown in Table 1. All devices achieved sealing pressure of 0.05 MPa with air as actuation media.

**Table 1:** Comparison of the sealing and rupture pressures of multi-layer microfluidic device with different PDMS ratios (N = 5).

PDMS ratios		Sealing pressure ( Mpa)	Rupture pressure
Flow layer	Control		
10:1	5:1	0.05	0.16–0.18 Mpa
20:1	5:1	0.05	> 0.24 Mpa (limit of pump)
20:1	10:1	0.05	> 0.24 Mpa (limit of pump)

The flow layer of the microfluidic devices used in the experimentation discussed above made use of about 50 µm thickness, this is to make it congruent to the reported device on a platform for single cell granzyme B profiling^20^. However, it should be noted that the thickness of the membrane separating the two layers is an important parameter for actuation pressure reduction. Considering its effect, in addition to the identified optimized geometry, devices with thinner flow layer were also fabricated. The results of the actuation test are summarized in the Table 2:

**Table 2:** Comparison of the sealing pressure of microfluidic devices with varying membrane thickness (N = 3).

RPM	Average flow layer thickness (µm)	Membrane thickness (µm)	Average sealing pressure (MPa)
2000	50	25–34	0.05
3000	36	15–23	0.05
3500	32	12–19	0.05
4000	27	–	0.04

Flow layers with thickness generated from spin coating PDMS at speeds of 2000 to 3500 RPM achieved the same mean sealing pressure with air as actuation medium. These resulted to membrane thickness of 12–19 µm, 15–23 µm, and 25–34 µm, respectively (Fig. S9). Meanwhile the thinnest membrane generated from a speed of 4000 RPM achieved the lowest sealing pressure of 0.04 MPa but many of the microstructures (i.e., pillars) are missing or not formed due to insufficient thickness. Also, air penetrating the membrane particularly within the closed microchamber, can be observed a minute after the start of actuation as shown in the supplementary video file (S10). This event could be possibly due to air permeating the thin membrane or a puncturing in the membrane. Nonetheless, the previous parameter (2000 RPM) was used in the fabrication of the microfluidic chip for the high-throughput enzymatic activity assay application.

### Single cell assay

The optimization conditions and fabrication customizations explored in this work were used in the fabrication of a high throughput valve-based microfluidic device that was utilized in the single cell enzymatic activity assay experiment. The purpose of this experiment is to quantify the expression of granzyme B protein from each cell and create a profile to show the variability of said expressions.

**Figure 6:**
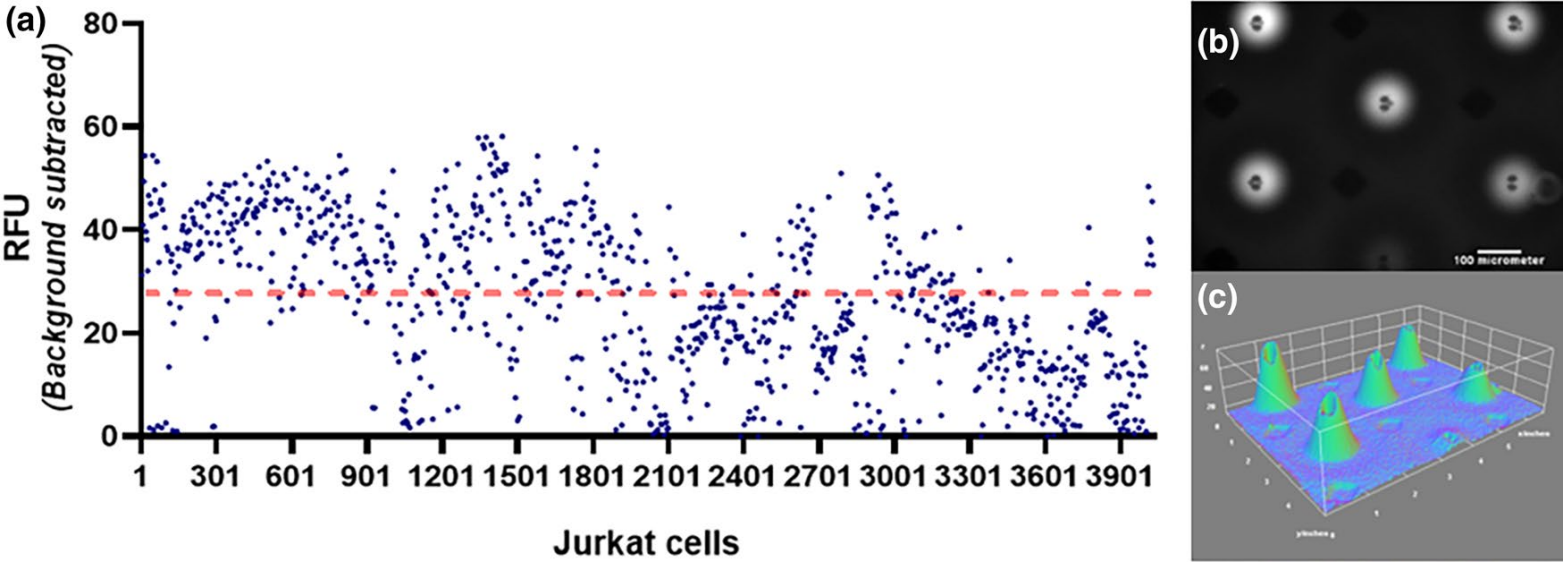
Single cell fluorometric granzyme B activity assay for jurkat cells (**a**) graph showing cell expression profile (dashed line indicates the mean value) (**b**) monochrome image of the microchambers showing the fluorescence (white) from AFC fluorophore (**c**) 3D surface plot of the same microchambers using ImageJ software. Graph 6a was created using GraphPad Prism 8.4.3. and 3D Plot 6c using ImageJ 1.52a.

After cell preparation, samples were introduced to the microfluidic device following experiment protocol (Fig. S6) About 3922 Jurkat cells, individually trapped and isolated in the microchambers, were assayed and its profile is shown in Fig. 6. With each data point representing individual cell, the graph shows the variability of protease expression at single cell level with a mean fluorescence intensity value of 27, a maximum value of 62, and a standard deviation of 14.6. The difference between the individual value was found significant using the One Sample Wilcoxon Test with a *P* value of < 0.0001 (alpha = 0.05).

**Figure 7:**
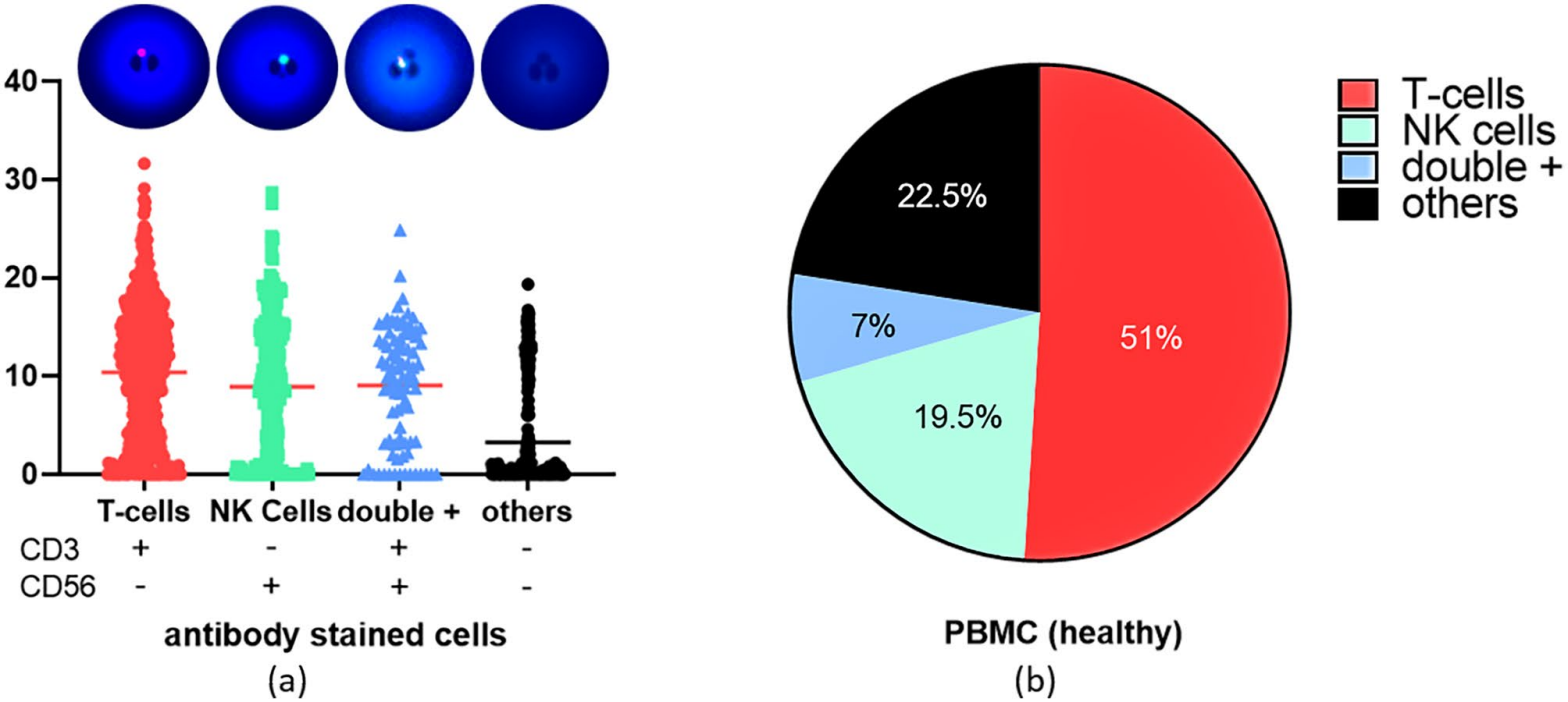
Single cell fluorometric granzyme B activity assay for PBMC cells from healthy person (**a**) antibody staining shows the immune cell composition and corresponding activity (red line indicates the mean value) (**b**) percent composition of immune cells in the profiled sample. Graph created using GraphPad Prism 8.4.3.

The same assay was also performed to PBMC cells from a healthy person (Fig. 7). The granzyme B activity at single cell level is found to be similarly varied with a mean value of 8, maximum of 39, and standard deviation of 7.2. A significant difference was also found using one sample Wilcoxon test with a *P* value of < 0.0001 (alpha = 0.05), demonstrating heterogeneity in the protein expression within the sample population. Only 2730 single cells were trapped for this test as PBMC cells have smaller diameter than Jurkat, allowing for some cells to escape through the trap gap. Figure 7a shows the GrB activity according to immune cell composition of the sample, identified via antibody immunostaining for CD3 (CD4 and CD8 expressing T cells), CD56 NK cells, and double positive (NKT cells). The percentage composition of the captured immune cells is shown in Fig. 7b.

The GrB profile for both the Jurkat and PBMC samples shows variability of protease expression in single cell level, which is important in monitoring heterogeneity and identification of cell subsets with overexpression. From the resulting profile, specific cells that are showing unique expression patterns can be identified. Other surface markers (i.e. anti-PD1, Ig-G4) can be introduced in the chip to determine the cell state or patient response to a particular treatment (i.e. immunotherapy). The information that can be gathered from the behavior of each cell, in general, or from individual immune cell, in particular, can lead to discoveries of several biological functions that are not observed in bulk analysis. This single cell analysis can provide information on genotype, phenotype, protein secretion, proliferation, maturation, activation, signaling pathways of each activated cell, and the intercellular communication among different immune cells^2,24^.

## Conclusion

In order to improve the throughput of the valve-based microfluidic device, optimization of the valve design and fabrication technique was sought. Design modeling through COMSOL Multiphysics was done to determine the valve dimension and geometry that could result to lower sealing pressure. Optimization in the fabrication technique was also pursued through the addition of a glass slip a few microns above the valve cavities in order to increase the stiffness and reduce the strain of the top portion. The effect of air and oil as actuation media to the sealing of the microchambers was also compared.

Applying these optimization conditions resulted to the fabrication of a microfluidic device composed of 5000 circular microvalves that can be actuated at a pressure as low as 0.04 MPa to seal all chambers. The reduction in the working pressure is seen significant as it presents a more reliable valve actuation and gives more room to increase the number of valves that can be added to the chip and thus further increasing the throughput. Higher throughput devices give a better representation of the sample population and is suited for single cell analysis experiments that investigate heterogeneities and identify unique cell subsets or super mutants from thousands of cells. The retrieval of these cells of interest can provide important information to researchers and clinicians, which can lead to drug discovery and improvement in treatment personalization.

## Methods

### Multiphysics modeling

The model formulated in this work was developed to describe the pneumatic operation of the microchamber block. The geometry and specifications shown in Figure S7a and Table S1 were used in modeling the microvalve, constructed using the COMSOL Multiphysics (ver5.5) simulation tool. Since the expression level of granzyme B per cell is low, the resulting chamber volume was kept to less than 100 picolitre in order to isolate a higher concentration of the biochemical agent^29,30^. Thus, the valve’s inner radius was set to 30 µm. Meanwhile, the valve’s outer radius (R), height (h), and overall structure was varied to gain insight into the dependence of the actuation/sealing pressure on the geometry of the valve.

The membrane deflection in the microchamber block of the microfluidic device was simulated using the laminar flow and solid mechanics modules. The laminar flow for fluid flow physics was used for valve channel in which stationary simulation for weakly compressible flow was carried out to obtain the fluid velocity and pressure profile. Air was used as the fluid for the simulation.

The solid mechanics for structural mechanics physics was used for the elastomer membrane deflection to determine the relation of the valve diameter and height to the sealing pressure. PDMS was modeled as a linear elastic material with elastic modulus of 2.63 MPa (for a 10:1 pre-polymer/curing agent ratio^22^), a Poisson’s ratio of 0.49 and a density of 950 kg/m^3^. The Neo-Hookean model was used for the hyperelastic, near-incompressible, material with Lamé parameter, µ, of 6.67 × 10^5^ N/m^2^ and bulk modulus, κ, of 3.33 × 10^7^ Pa. This constitutive model describes the behavior of a rubber-like material undergoing large deformation which is obtained by a statistical-mechanical deformation treatment of freely jointed molecular chains. A glass block was placed at the bottom part of the chamber model, representing a glass substrate, and set as a fixed constraint. All other material properties for the simulation were taken from the COMSOL material library. The physics-controlled finer mesh was used and is shown in Figure S7b.

### Device fabrication

Identified optimal parameters from the simulation analysis were applied in the device fabrication. To fabricate and assemble the microfluidic device, two molds on a 4” silicon wafers were created, following its corresponding AutoCAD design, using optical photolithography. For the flow layer mold, SU8 3010 and 3025 photoresists (MicroChem, Massachusetts, USA) were spin coated (1H-D7, Mikasa Co. Inc., Japan) at different steps to fabricate structures of varying heights (15 µm and 25 µm). Meanwhile, SU8 3025 was used for the control layer mold following the same steps to create microstructures of 25 µm and 50 µm. The resists were then exposed to UV light source (20,000 mJ cm^−2^ at 405 nm) using a maskless exposure apparatus (Nano System Solutions DL-1000). The molds were then developed and subsequently silanized with 1H,1H,2H,2H-perfluorodecyltrichlorosilane (Wako Pure Chemical Industries, Ltd.) for ease of release of PDMS from the mold. Figures S4a and S4b shows the AutoCAD design of the flow and control layers.

An outline of the fabrication steps for mold fabrication and PDMS device assembly is shown in Fig. S1 and S2 of the supplementary material. The microfluidic chip was then created through pattern transfer using PDMS. A 10:1 ratio of PDMS oligomer and curing agent (Sylgard 184 silicone elastomer kit, Dow Corning, USA) was spin coated (1H-D7, Mikasa Co. Inc., Japan) on the flow layer mold to produce about 50 µm (2000 RPM, 30 s spin time) thick PDMS layer. Meanwhile, a 5:1 ratio was coated on the control layer mold to create a thicker layer. After partially curing at 80 °C for 30 min, the two PDMS layers were peeled, cut to size, and aligned. Since the elastomer flow layer is very thin to be handled without damage, the thick control layer is first removed from its mold and aligned on top of the flow layer. The remaining 5:1 PDMS mixture was poured around the aligned layers and then cured for 2 h at 80 °C. Here, excess functional groups in the two layers interact to form a covalent bond across the interface, resulting to an irreversible bonding of the two layers^28^. The PDMS chip was finally bonded to a cleaned microscopy coverslip after exposure to oxygen plasma for 50 s in a Plasma Dry Cleaner (Yamato PDC210) at maximum power (75 W). The resulting microfluidic device is shown in figure S4c. The thin flow layer at the bottom has microstructures that serves as hydrodynamic traps for single cells while the thick control layer on top has circular pneumatic microvalves that can be actuated to create a sealed microchamber (Fig. S4d and S4e).

### Valve actuation and chamber sealing

To form a sealed microchamber with a trapped cell inside, we made use of a circular microvalve design and actuate it with positive pressure using an air pump (ULVAC DA-60S). This shape was chosen as rectangular valves can cause leak at the corners of the channel valve intersection (Fig. S3). Microfluidic valves are usually formed when the control channel crosses the fluid channel. In a square or rectangular profile, the gaps created at incompletely closed corners during actuation are large enough to allow fluids to pass^31^. A comparison of an actuated circular and rectangular valve is shown in the supplementary material.

A sealed microchamber was determined by filling the flow channels with fluorescent solution FITC dextran (1 mg ml^−1^, Sigma-Aldrich) and actuating the valves afterwards (Fig. S5). The fluorescence at the actuated valve area of the chamber is measured and compared to the intensities of the areas without valve actuation under varying applied pressure. The effect of varying conditions (i.e. structure and actuation media) to the sealing pressure was compared.

### Device operation and single cell assay

The microfluidic device was used to conduct single cell assay using Jurkat T-lymphocyte cell and human peripheral blood mononuclear cell (PBMC). Granzyme B (GrB)-overexpressing Jurkat cells, provided by the collaborators from Osaka University Hospital, were generated by lentiviral transduction of expression plasmid. The cell samples were expanded from a cryopreserved master cell bank and maintained in a 10% fetal bovine serum (Gibco FBS, Thermo Fisher Scientific) medium and 1% penicillin in RPMI (Gibco RPMI 1640, Thermo Fisher Scientific). The cultures were stored in an incubator at 37 °C and 5% CO_2_ prior to conducting the assay experiment. The handling and experiments on cell samples were performed in accordance with the protocols of the Research Safety Committee of Osaka University.

To reduce cell adhesion on the channel walls, the microfluidic chip was blocked with 1% BSA (bovine serum albumin, Sigma-Aldrich, USA) in PBS solution for 1 h. Using a syringe pump, the chip was washed with PBS for 10 min at a flow rate of 50 µL min^−1^. In the conduct of the assay experiment, a cell suspension was flushed into the chip at a flow rate of 30 µL min^−1^ to be captured by the hydrodynamic traps. Excess cells were flushed out by rinsing the chip with 50 µL of buffer medium. Afterwards, the control channels and microvalves were actuated to seal the microchambers and isolate each trapped cell. A granzyme B recognition substrate (Ac-IEPD-AFC) in buffer from a commercial assay kit (Promocell GmbH, Heidelberg, Germany) was introduced into the microchambers by briefly opening and closing the valves. After 30 minutes incubation, the expressed GrB molecules from individual cells were quantified fluorometrically through the cleaving of the peptide substrate. A fluorescence microscope (BZ-X810, Keyence; 2/3 inch, 2.83 million-pixel monochrome CCD camera) was used to observe and capture the images of the microchambers. PH and DAPI (360/460 nm) filters were used during image acquisition. The acquired images were then analyzed and quantified for its fluorescence intensity using ImageJ image analysis software.

Immunostaining was also performed to PBMC cells for cell surface markers CD3 (CD4 and CD8 expressing T cells, Alexa Fluor 532) and CD56 Natural Killer (NK) cells (Alexa Fluor 488). This is to identify the immune cell composition of the sample. An outline of the single cell assay experiment can be found in the supplementary material (Fig. S6).

## Supplementary Information

Supplementary Information 1.

Supplementary Video 1.
