# Supplementary material for: A design and optimization of a high throughput valve based microfluidic device for single cell compartmentalization and analysis
Source: Sci Rep. 2021 Jun 21;11:12995. doi: 10.1038/s41598-021-92472-w (PMC8217553; doi:10.1038/s41598-021-92472-w)
Supplement: Supplementary file 1 — Supplementary Information 1. [file 41598_2021_92472_MOESM1_ESM.pdf]

SUPPLEMENTARY MATERIAL

A design and optimization of a high throughput valve based microfluidic device for single cell compartmentalization and analysis

Jonathan Briones [1], Wilfred Espulgar [1], Shohei Koyama[2], Hyota Takamatsu[2], Eiichi Tamiya[3,4], and Masato Saito [1,3]*

[1] *Graduate School of Engineering, Osaka University, Suita, Osaka 565-0871, Japan*

[2] *Graduate School of Medicine, Osaka University, Suita, Osaka 565-0871, Japan*

[3] *AIST PhotoBIO-OIL, Osaka University, Suita, Osaka 565-0871, Japan*

[3] *The Institute of Scientific and Industrial Research, Osaka University, Suita, Osaka 565-0871, Japan*

*Author to whom correspondence should be addressed. Electronic mail: saitomasato@ap.eng.osaka-u.ac.jp

I. Fabrication of master molds for PDMS casting and device assembly

A. Mold fabrication steps

↓

Silicon wafer (4") preparation
Dehydration using 95℃ hot plate

↓

Spin coat (1H-D7, Mikasa Co. Inc., Japan) SU8 photoresist
Different RPM & photoresist type (SU8 3010/ 3025, MicroChem USA) for different heights of microstructures

↓

Pre-exposure bake
60℃ (1 min) & 95℃ (time varies according to thickness)

↓

Multi-layer UV-exposure with maskless exposure apparatus following CAD design
(20,000 mJ cm^{-2} at 405 nm, Nano System Solutions DL-100)
*each mold is composed of 2 layers

↓

|Post exposure bake
60℃ (1 min) & 95℃ (time varies according to thickness)

↓

Development using SU8 developer (MicroChem, Massachusetts, USA)
*development time varies according to coating thickness

↓

Hard bake for 30 mins at 150 °C

↓

Silanization for 5 minutes
1H,1H,2H,2H-perfluorodecyltrichlorosilane (Wako Pure Chemical Industries, Ltd.)

↓

Installation of metal posts (eyelets) for inlet/outlet

Figure S1. Outline of the mold fabrication steps

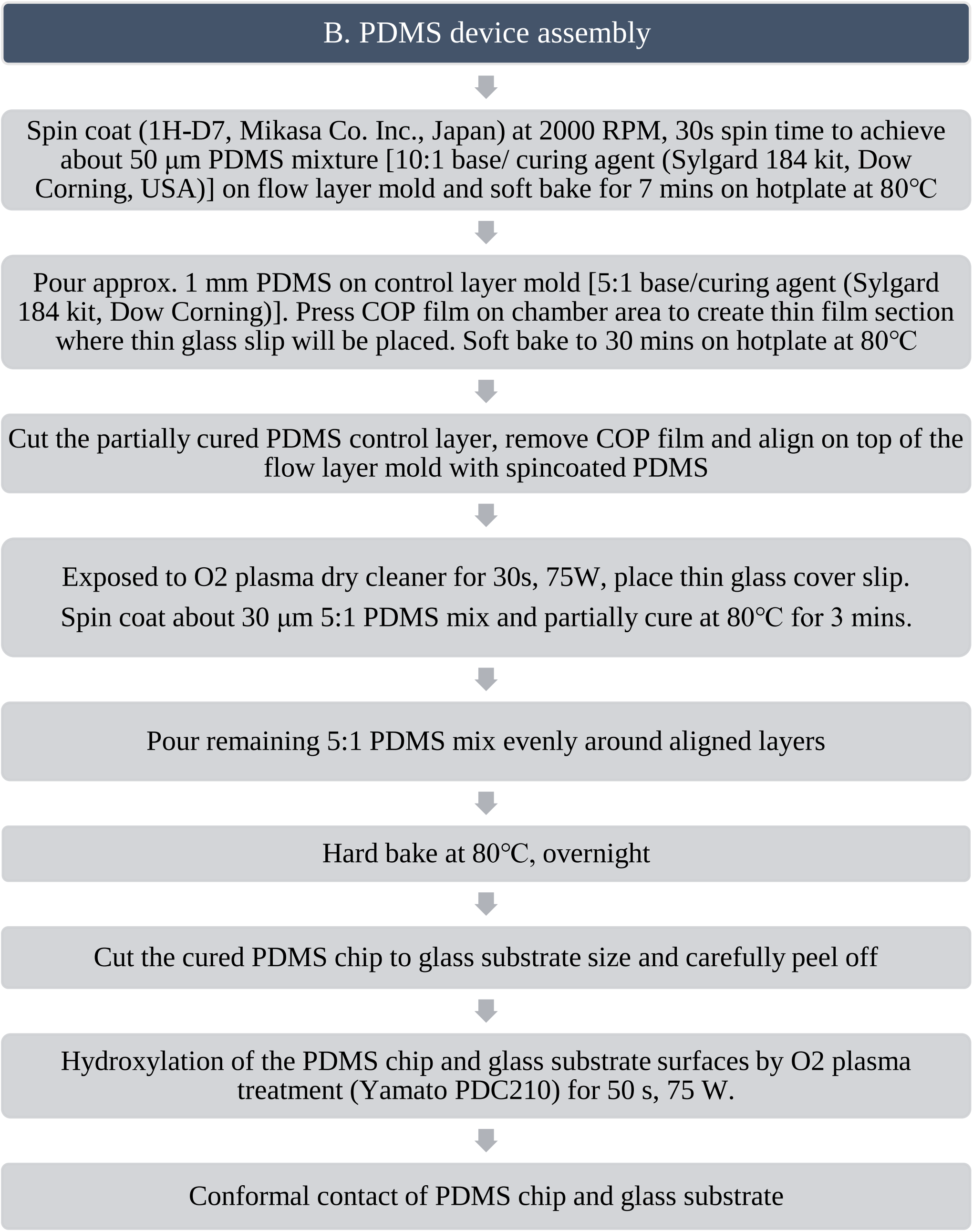

Figure S2. Outline of the microfluidic device assembly

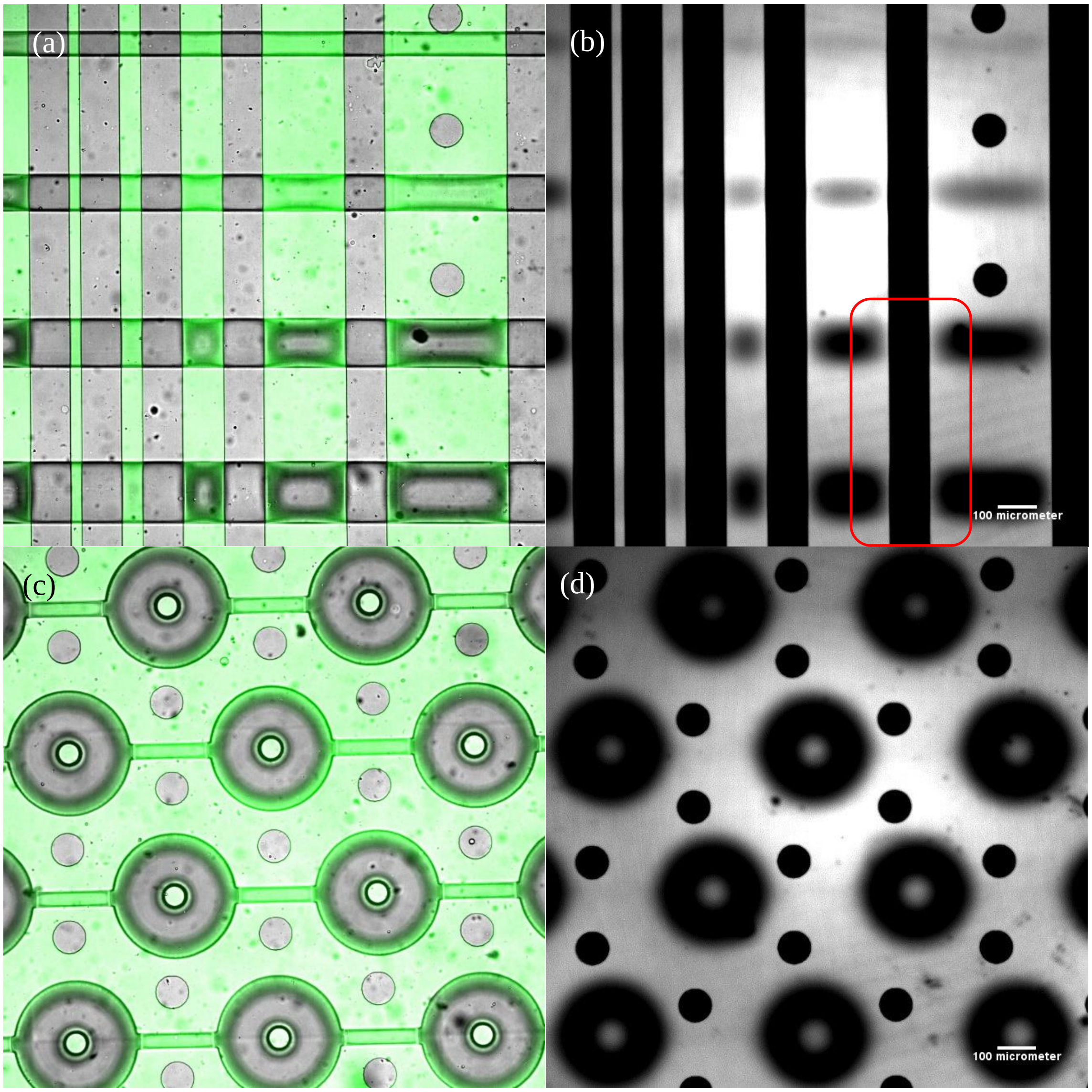

Figure S3. Microscope images of fluorescent solution filled microfluidic channels with air actuated valves at 0.14 MPa (a) multicolor (merged) image of the channels and actuated rectangular valves (b) monochrome (488 filter) showing the gaps present at incompletely closed corners (c) multicolor (merged) image of the channels and actuated circular valves (d) monochrome (488 filter) image of sealed microchambers.

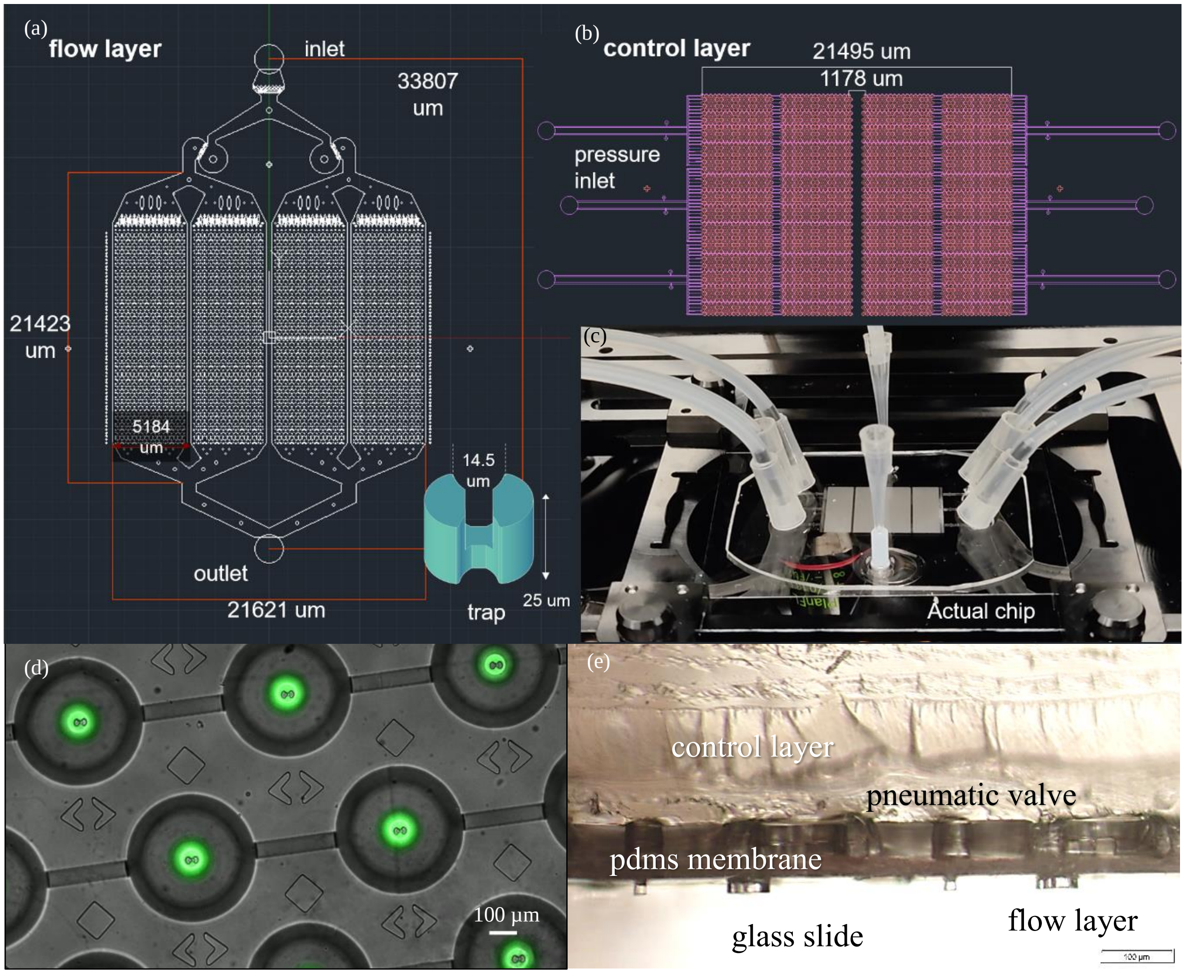

Figure S4. Microfluidic chip design for high throughput single cell assay (a) CAD design of the flow layer (inset) hydrodynamic trap design (b) CAD design of the control layer (c) photo of the assembled microfluidic device (d) microscope image of an array of actuated valves confining a fluorescent solution (e) microscope image of the chip's cross-section. Figures a and b created using Autodesk AutoCAD 2021 student version R.47.0.0.

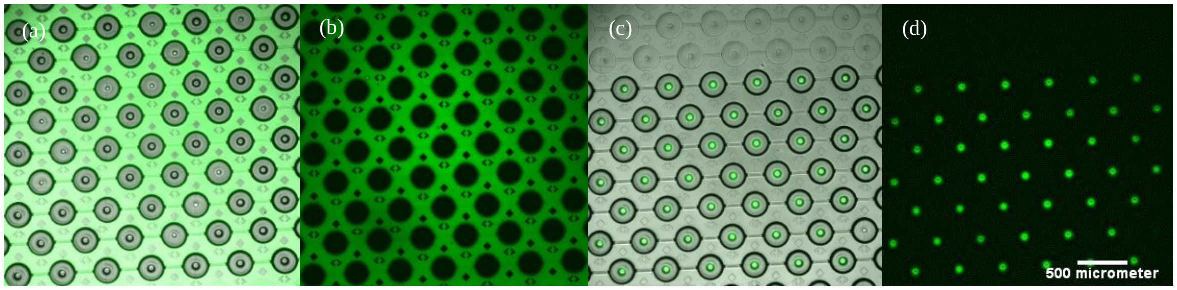

Figure S5. FITC dextran fluorescence solution was used to check the sealing of the microchambers. The solution was made to flow into the channels and the microvalves were actuated (a) multicolor image of the channel and valve array (b) image under FITC (488) filter. After opening-closing the microvalves, the channel was washed by flowing PBS while the valves are actuated leaving the fluorescent solution trap inside the chamber (c) multicolor image (d) image under FITC (488) filter.

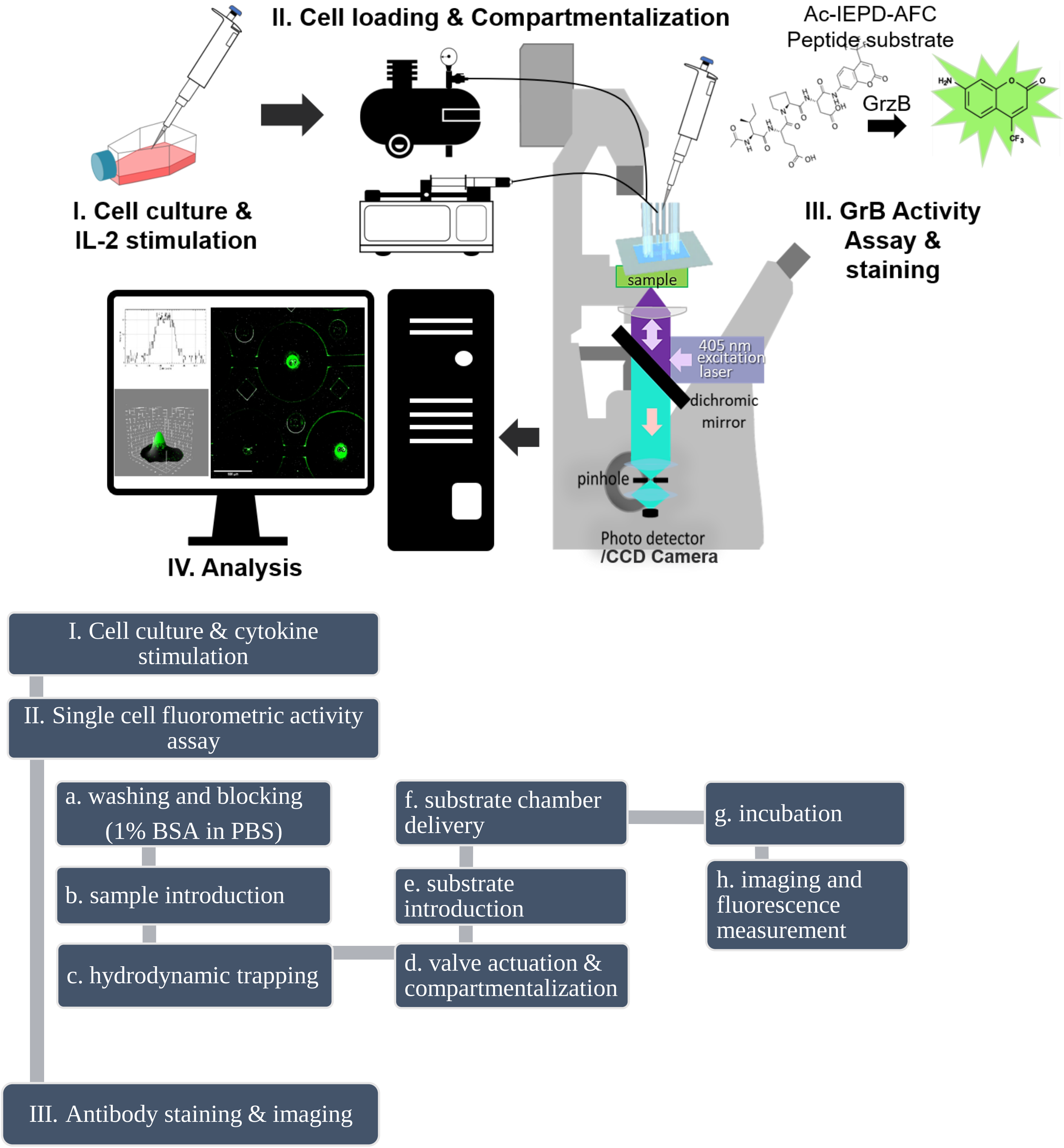

Figure S6. Illustration depicting the steps of microfluidic device operation for single cell fluorometric activity assay.

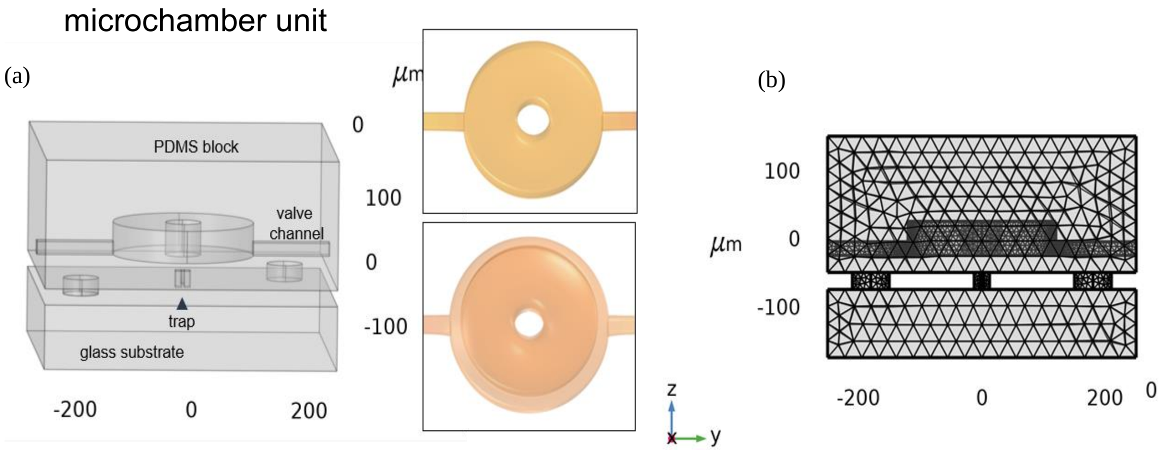

Figure S7. Microfluidic chamber block comprising of the control microvalve and mechanical trap (a) COMSOL rendered valve geometries (b) physics-controlled finer mesh model. Figures were generated using COMSOL Multiphysics 5.5 software.

Table S1. Specifications of the microvalve used for simulation

<table>
<tr><th>valve unit</th><th>inner radius, r (µm)</th><th>outer radius, R (µm)</th><th>height (µm)</th></tr>
<tr><td>A</td><td rowspan="8">30</td><td>80</td><td rowspan="4">25</td></tr>
<tr><td>B</td><td>100</td></tr>
<tr><td>C</td><td>120</td></tr>
<tr><td>D</td><td>140</td></tr>
<tr><td>E</td><td>80</td><td rowspan="4">50</td></tr>
<tr><td>F</td><td>100</td></tr>
<tr><td>G</td><td>120</td></tr>
<tr><td>H</td><td>140</td></tr>
</table>

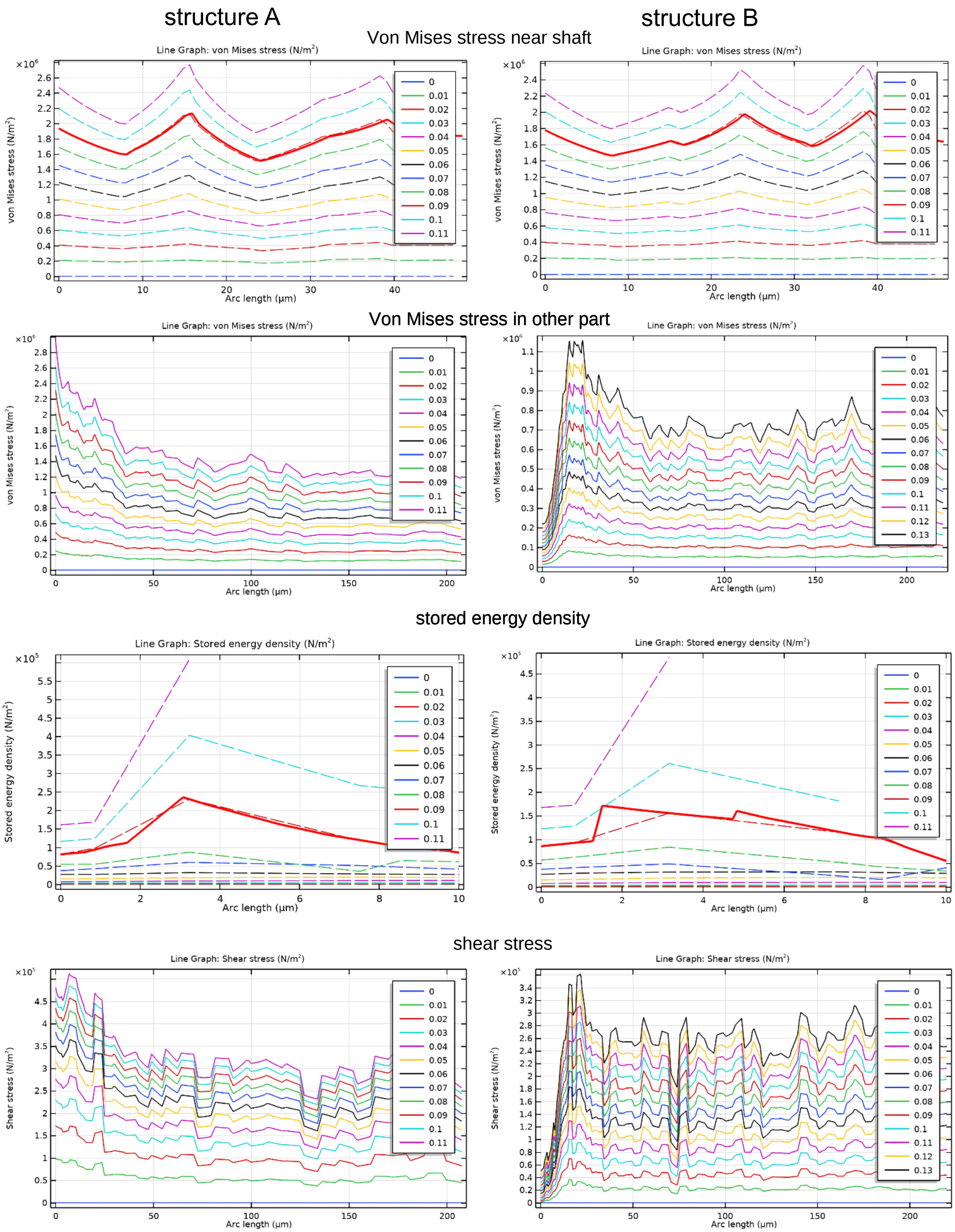

Figure S8. Comparison of the von Mises stress, total stored or strain energy density, and shear stress in structures A and B. Figures were generated using COMSOL Multiphysics 5.5 software.

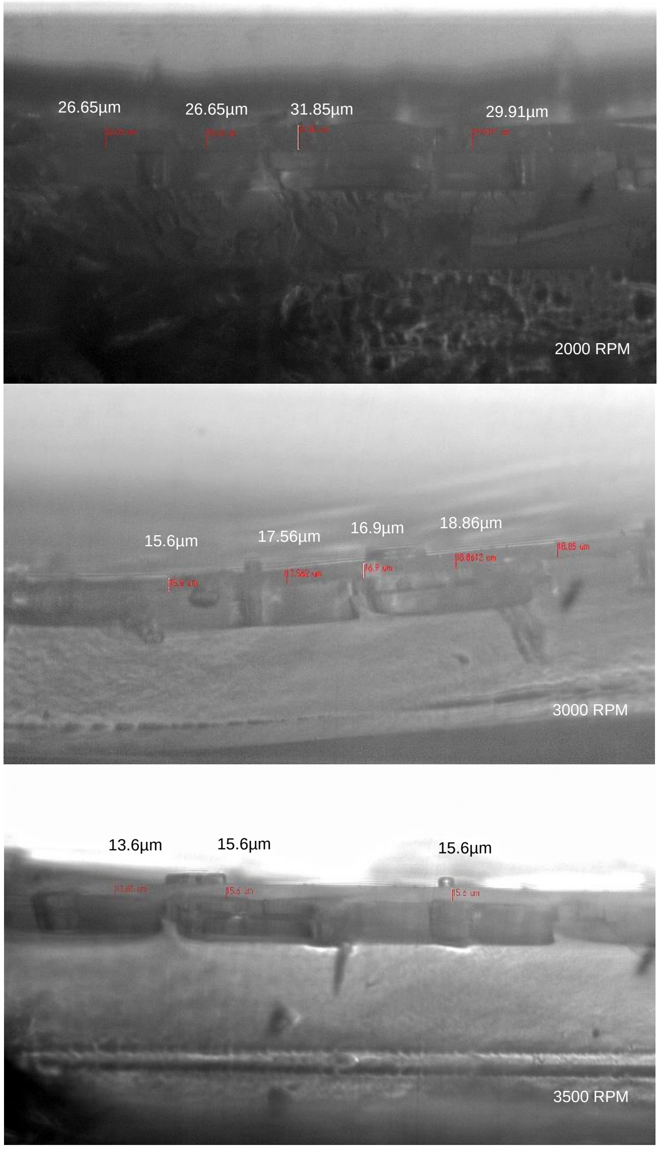

Figure S9. Measurement of the PDMS membrane thickness resulting from varying spin coating RPM

File S10. Video of actuated microchambers with gas (bubbles) emanating within the microchamber.
